# Supplementary material for: Chitosan or Cyclodextrin Grafted with Oleic Acid Self-Assemble into Stabilized Polymeric Micelles with Potential of Drug Carriers
Source: Life (Basel). 2023 Feb 4;13(2):446. doi: 10.3390/life13020446 (PMC9964696; doi:10.3390/life13020446)
Supplement: Supplementary file 1 [file life-13-00446-s001.zip › Supplement.pdf]

Supplementary Materials

Polymeric micelles based on chitosan and cyclodextrin, grafted with oleic acid for improved solubility and delivery of antibacterial agents in the bacterial cells

Igor D. Zlotnikov [1,*], Dmitriy A. Streltsov [1], Natalya G. Belogurova [1] and Elena V. Kudryashova [1,*]

[1] Faculty of Chemistry, Lomonosov Moscow State University, Leninskie Gory, 1/3, 119991 Moscow, Russia;

* Correspondence: helenakoudriachova@yandex.ru (E.V.K.), zlotnikovid@my.msu.ru (I.D.Z.)

Content

Figure S1. Particle size distribution by NTA: (a) amCD-OA, (b) Chit5-OA-5, (c) Chit5-OA-15, (d) Chit5-OA-30. T = 22 C. MilliQ H_2O. 10^9-10^{10} particles/mL. (e) TNBS spectrophotometric titration curves of chitosan, amCD and conjugates NH_2-group. T = 22 C. 0.02 M $Na_2B_4O_7$ (pH 9.2)

Figure S2. Fluorescence determination of the critical concentration of micelle formation of amCD-OA. (a) Normalized fluorescence emission spectra of pyrene label. (b) Pyrene fluorescence anisotropy spectra. (c) Peaks intensities of the spectra (a) on the concentration of amCD-OA dependences. The intersection point roughly corresponds to the CMC. PBS (0.01M, pH 7.4). λ_{exci}(pyrene) = 340 nm. T = 22 °C.

Figure S3. Fluorescence determination of the critical concentration of micelle formation of Chit5-OA-5. (a) Normalized fluorescence emission spectra of pyrene label. (b) Pyrene fluorescence anisotropy spectra. (c) Peaks intensities of the spectra (a) on the concentration of Chit5-OA-5 dependences. The intersection point roughly corresponds to the CMC. PBS (0.01M, pH 7.4). λ_{exci}(pyrene) = 340 nm. T = 22 °C.

Figure S4. (a) FTIR spectra of moxifloxacin (MF) and its loaded form into Chit5-OA-15 micelles. (b) FTIR spectra of moxifloxacin (MF) and its loaded form into Chit5-OA-30 micelles. 0.01M PBS (pH 7.4). T = 22 °C

Figure S1. Particle size distribution by NTA: (a) amCD-OA, (b) Chit5-OA-5, (c) Chit5-OA-15, (d) Chit5-OA-30. T = 22 C. MilliQ H_2O. 10^9-10^{10} particles/mL. (e) TNBS spectrophotometric titration curves of chitosan, amCD and conjugates NH_2-group. T = 22 C. 0.02 M $Na_2B_4O_7$ (pH 9.2)

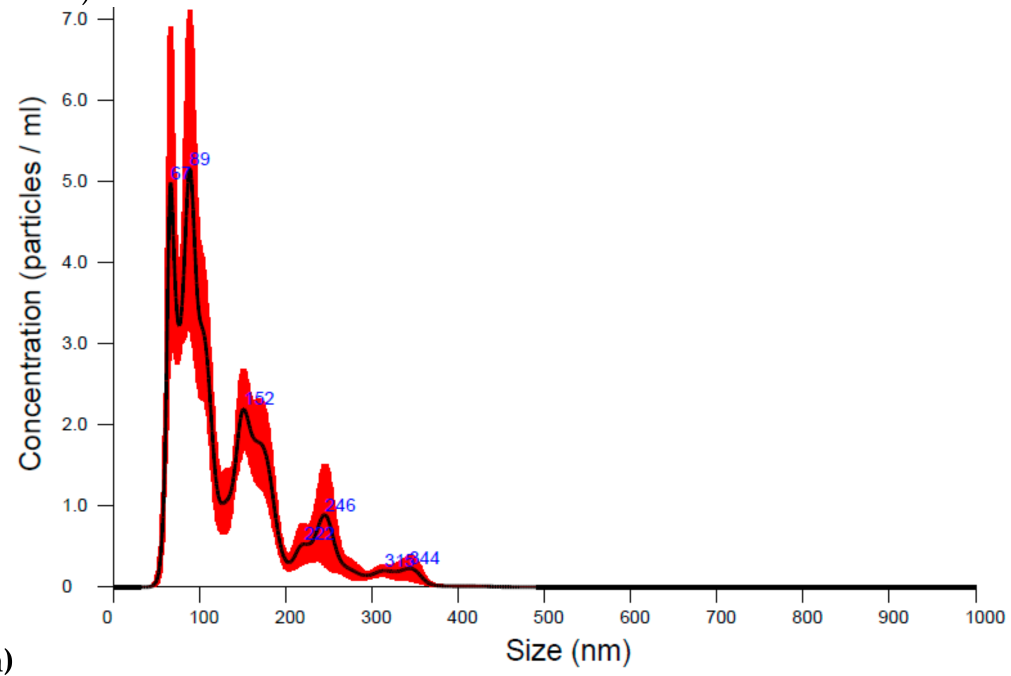

(a)

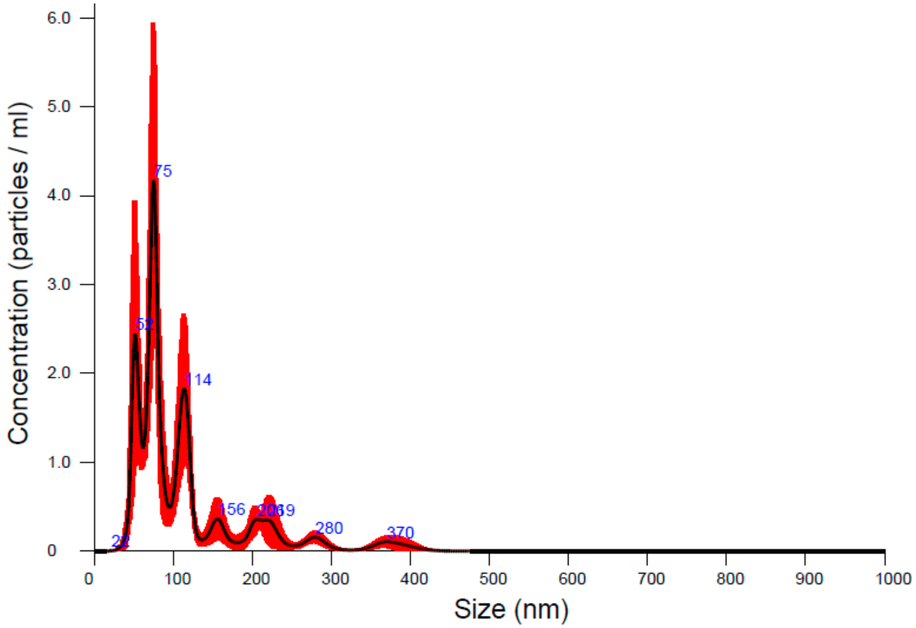
Concentration (particles / ml)
Size (nm)
0
1.0
2.0
3.0
4.0
5.0
6.0
0
100
200
300
400
500
600
700
800
900
1000
75
52
114
156
280
370

(b)

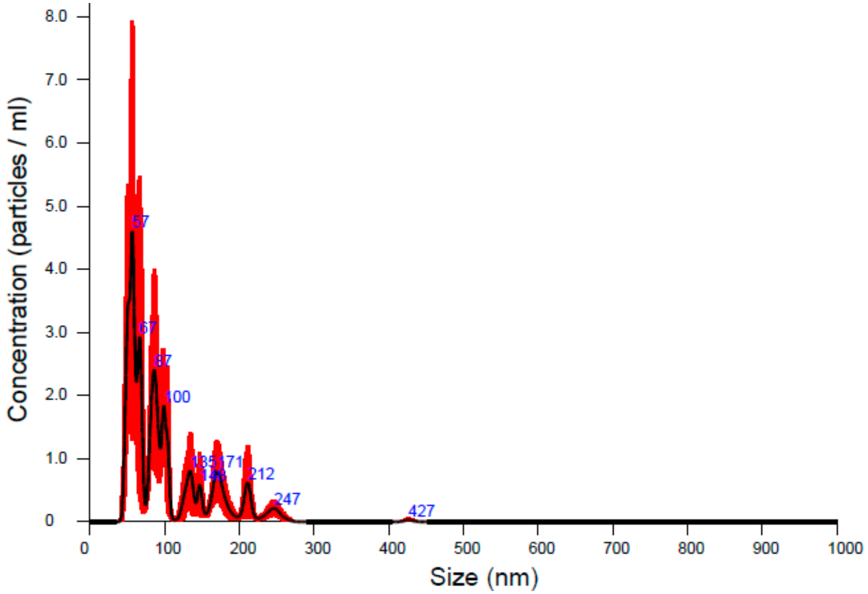
Concentration (particles / ml)
Size (nm)
0
1.0
2.0
3.0
4.0
5.0
6.0
7.0
8.0
0
100
200
300
400
500
600
700
800
900
1000
57
67
87
100
212
247
427

(c)

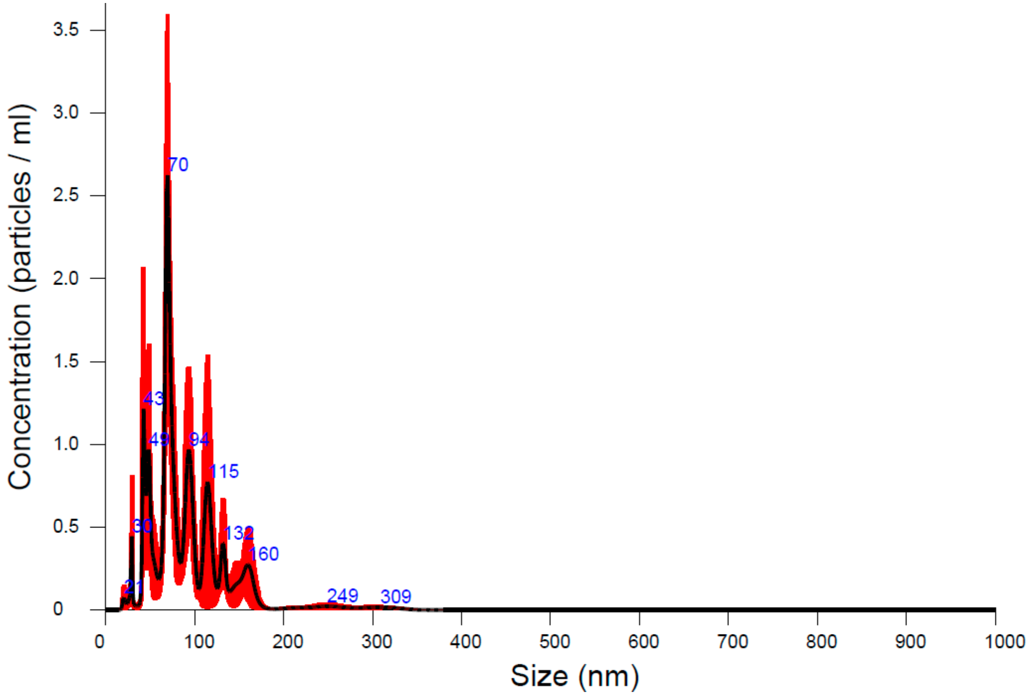
Concentration (particles / ml)
3.5
3.0
2.5
2.0
1.5
1.0
0.5
0
70
43
49
94
115
30
132
160
249
309
0
100
200
300
400
500
600
700
800
900
1000
Size (nm)

(d)

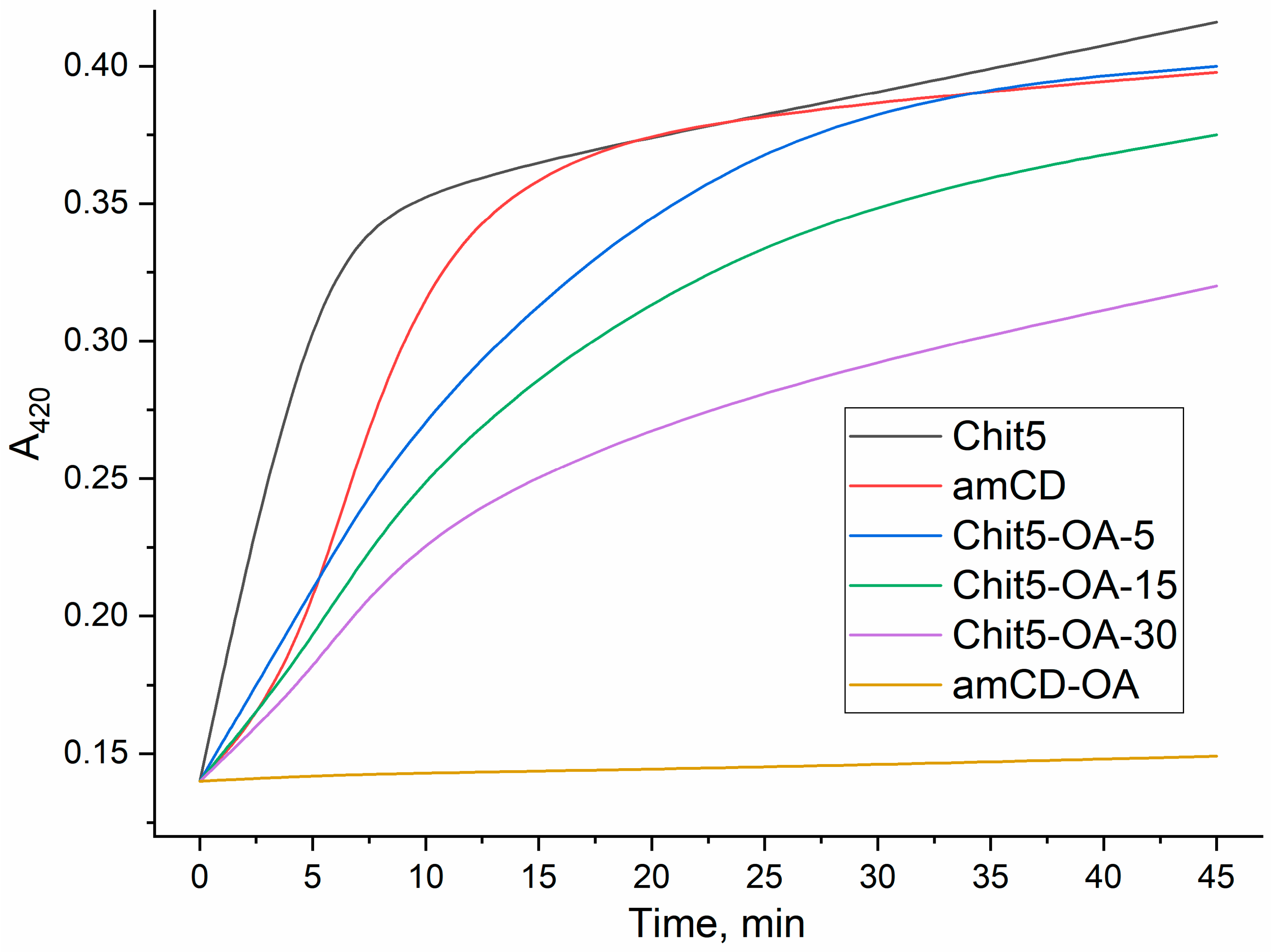

A_{420}
0.40
0.35
0.30
0.25
0.20
0.15
0
5
10
15
20
25
30
35
40
45
Time, min
Chit5
amCD
Chit5-OA-5
Chit5-OA-15
Chit5-OA-30
amCD-OA

(e)

Figure S2. Fluorescence determination of the critical concentration of micelle formation of amCD-OA. (a) Normalized fluorescence emission spectra of pyrene label. (b) Pyrene fluorescence anisotropy spectra. (c) Peaks intensities of the spectra (a) on the concentration of amCD-OA dependences. The intersection point roughly corresponds to the CMC. PBS (0.01M, pH 7.4). λ_{exci}(pyrene) = 340 nm. T = 22 °C.

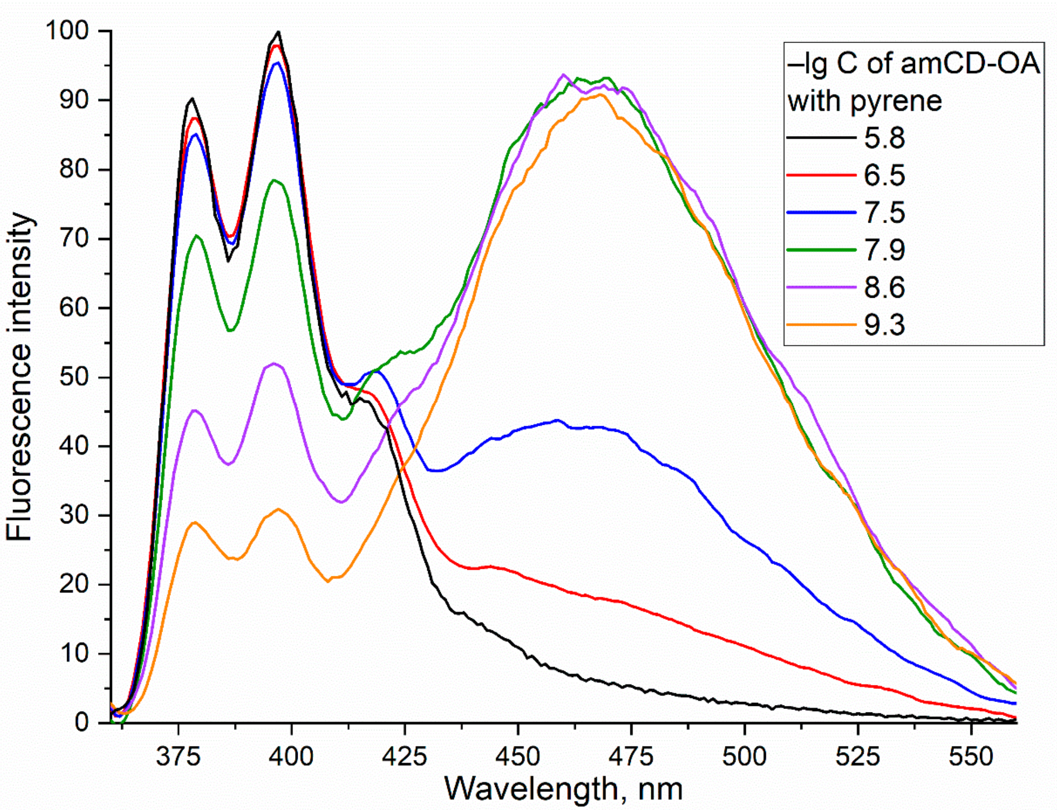

(a)

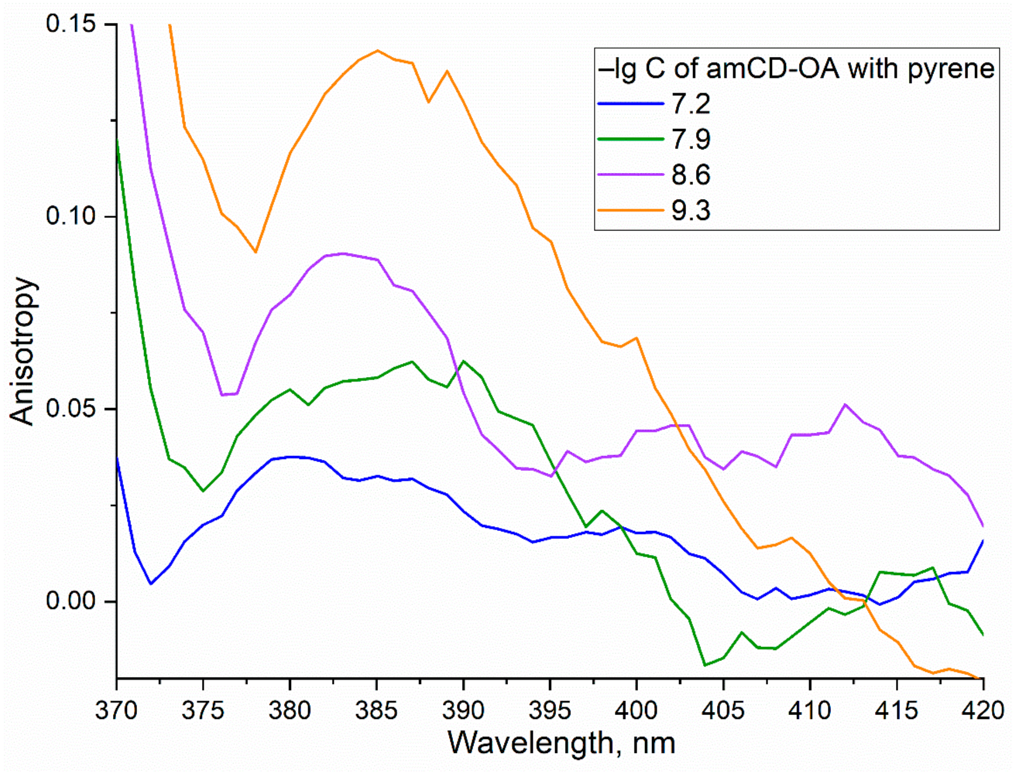
0.15
0.10
0.05
0.00
Anisotropy
370
375
380
385
390
395
400
405
410
415
420
Wavelength, nm
–lg C of amCD-OA with pyrene
7.2
7.9
8.6
9.3

(b)

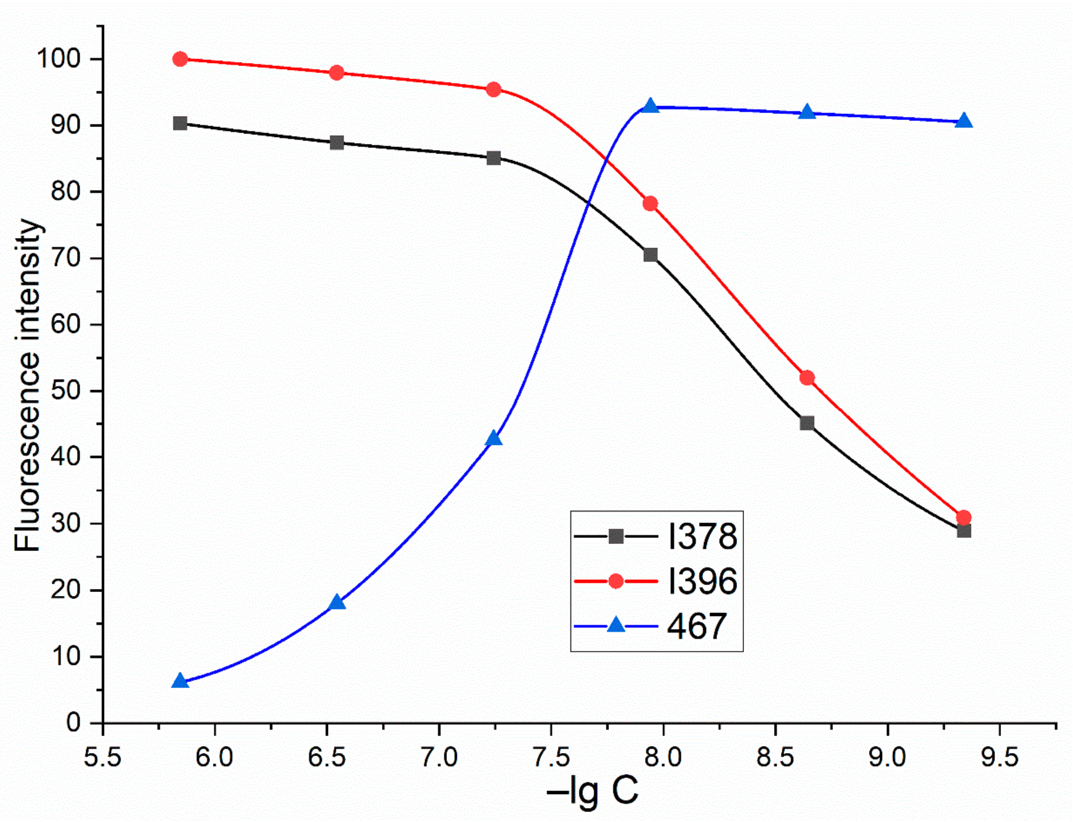

(c)

Figure S3. Fluorescence determination of the critical concentration of micelle formation of Chit5-OA-5. (a) Normalized fluorescence emission spectra of pyrene label. (b) Pyrene fluorescence anisotropy spectra. (c) Peaks intensities of the spectra (a) on the concentration of Chit5-OA-5 dependences. The intersection point roughly corresponds to the CMC. PBS (0.01M, pH 7.4). λ_{exci}(pyrene) = 340 nm. T = 22 °C.

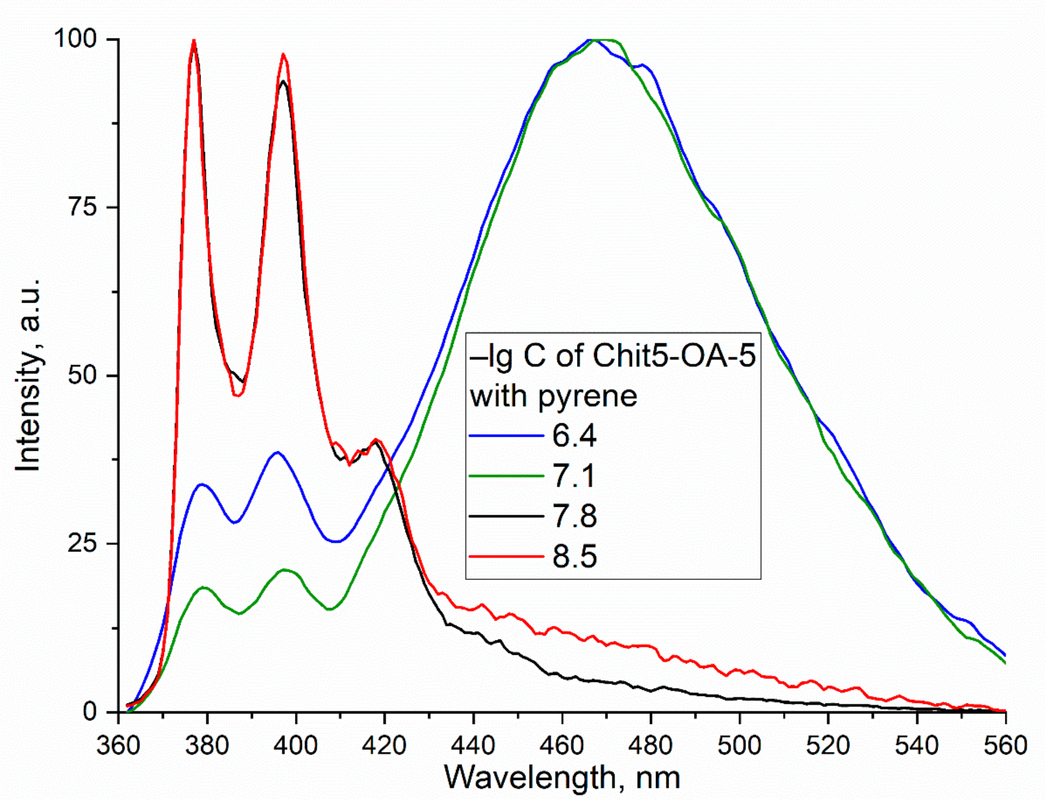

(a)

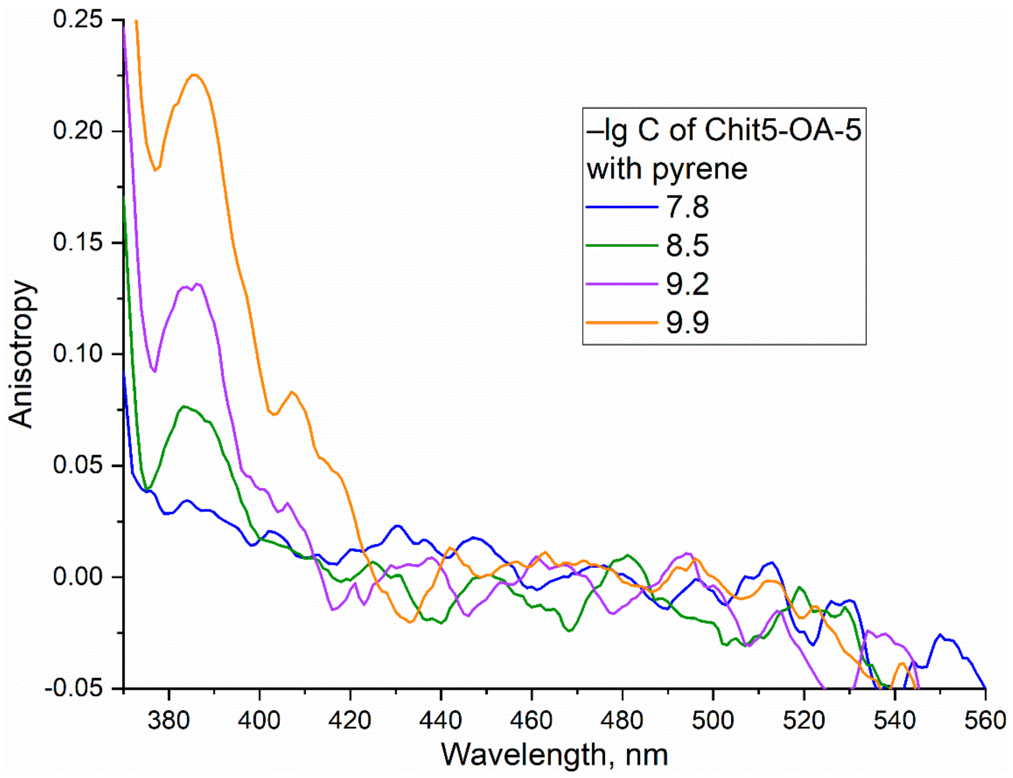

0.25
0.20
0.15
0.10
0.05
0.00
-0.05
Anisotropy
380
400
420
440
460
480
500
520
540
560
Wavelength, nm
–lg C of Chit5-OA-5
with pyrene
7.8
8.5
9.2
9.9

(b)

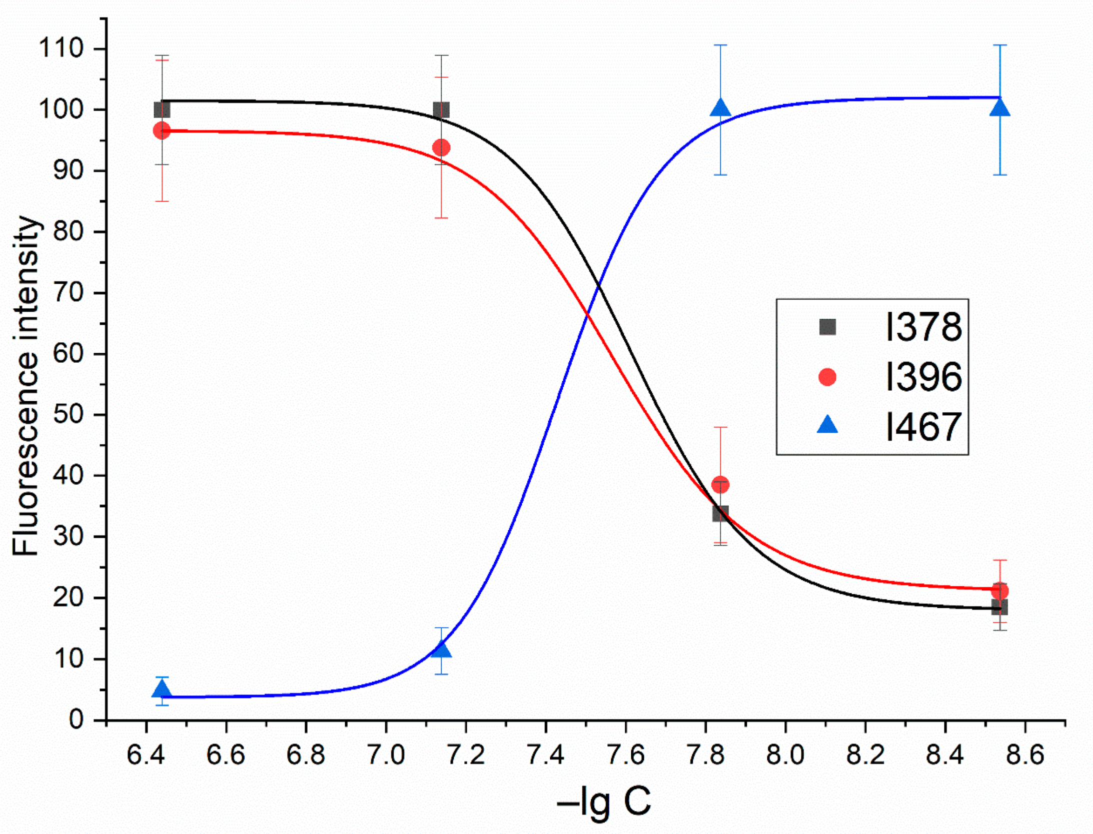

Fluorescence intensity
110
100
90
80
70
60
50
40
30
20
10
0
6.4
6.6
6.8
7.0
7.2
7.4
7.6
7.8
8.0
8.2
8.4
8.6
–lg C
I378
I396
I467

(c)

Figure S4. (a) FTIR spectra of moxifloxacin (MF) and its loaded form into Chit5-OA-15 micelles. (b) FTIR spectra of moxifloxacin (MF) and its loaded form into Chit5-OA-30 micelles. 0.01M PBS (pH 7.4). T = 22 °C

(a)

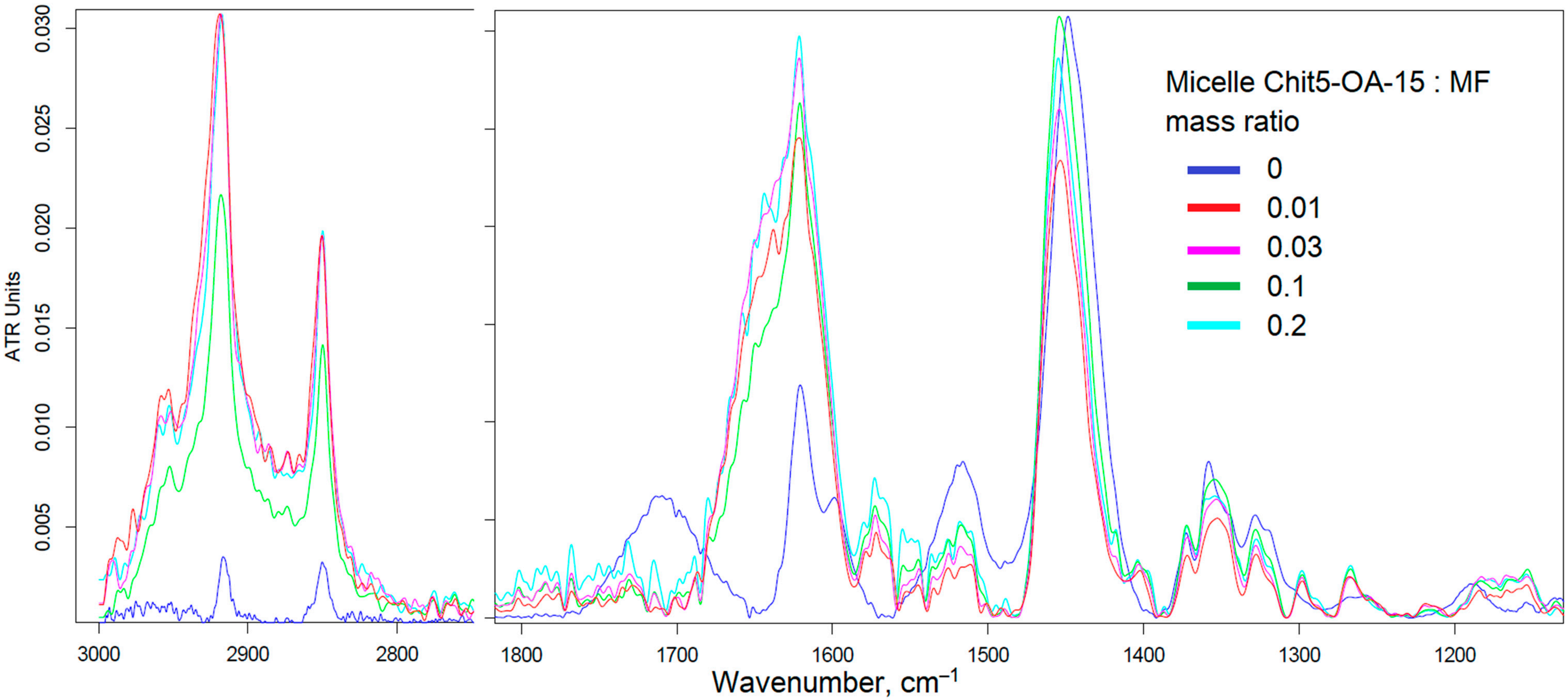

(b)

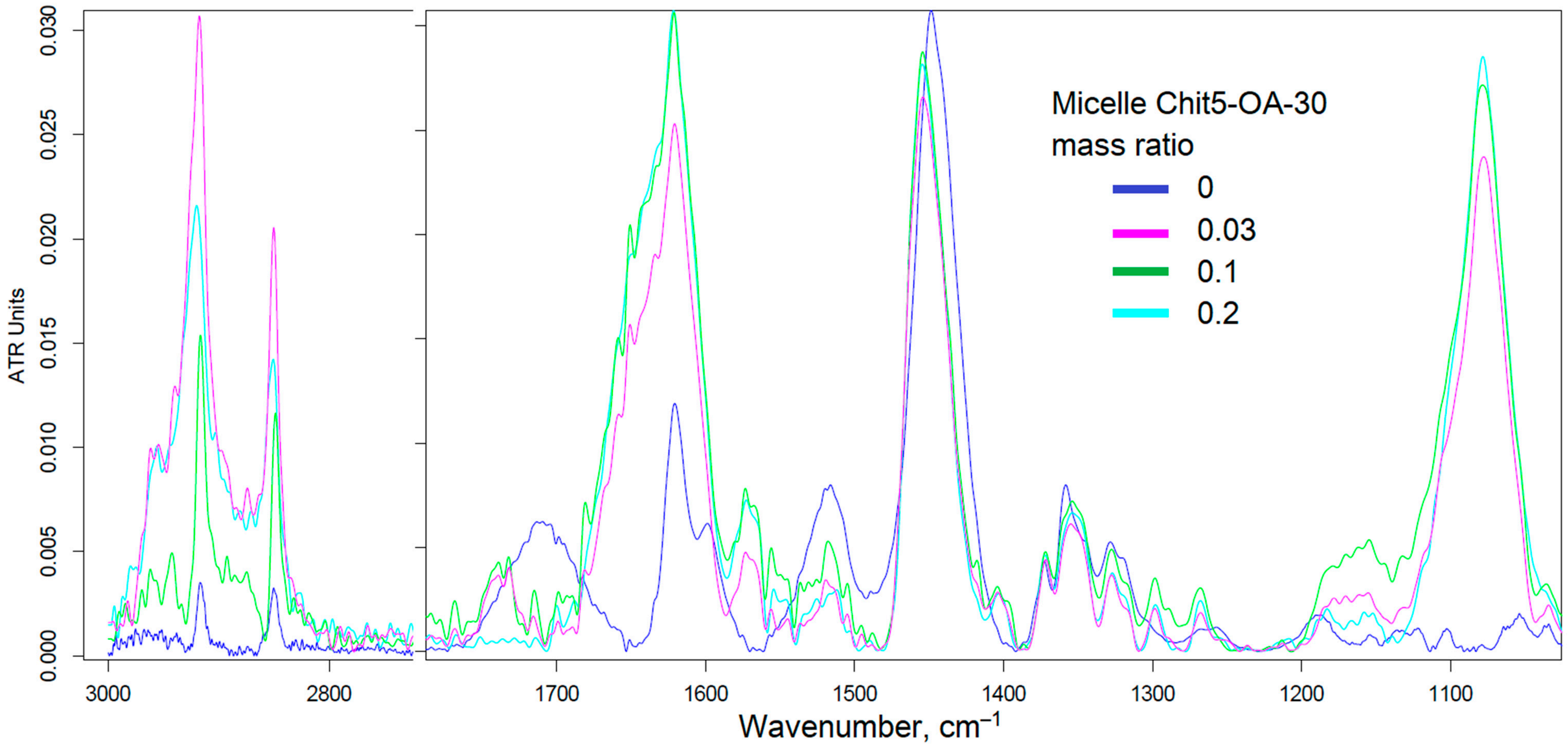

ATR Units
0.000
0.005
0.010
0.015
0.020
0.025
0.030
3000
2800
1700
1600
1500
1400
1300
1200
1100
Wavenumber, cm^{-1}
Micelle Chit5-OA-30
mass ratio
0
0.03
0.1
0.2
